# Signaling networks, network pathology and computational chemotherapy

**DOI:** 10.18632/oncotarget.911

**Published:** 2013-02-27

**Authors:** Kwang-Hyun Cho

**Affiliations:** Department of Bio and Brain Engineering, Korea Advanced Institute of Science and Technology (KAIST), Daejeon, Republic of Korea

**Figure 1:**
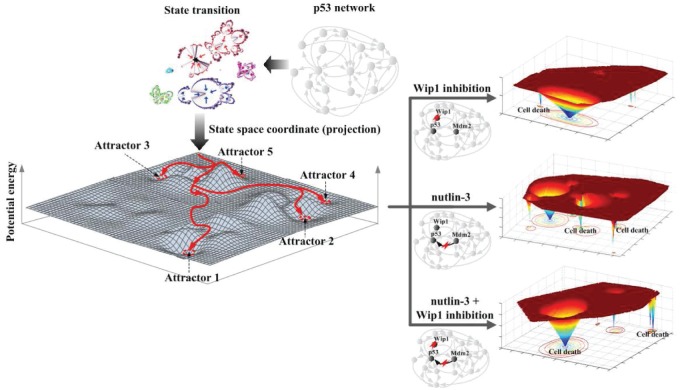
The state space analysis of the p53 network and the resulting potential energy landscapes for MCF7 cells after different inhibitory treatments that modify the network and induce cell death in the presence of DNA damage Attractor landscape analysis showing the state transition dynamics upon the potential energy landscape of p53 network is illustrated (left). For the p53 network of MCF7 cells, the treatment of nutlin-3 in combination with inhibition of Wip1 (right bottom) resulted in a synergistic effect in producing much larger basins of cell death attractors compared to the single treatment of either Wip1 inhibition (right upper) or nutlin-3 (right middle).

Cellular signal transduction pathways are highly interconnected and form a complex network where many signaling molecules interact with each other in a complicated manner [1-2]. The complexity of the network structure suggests that the signal transduction network may conduct sophisticated information processing and decision making for cell fate determination rather than simple passing of an outer signal to the interior of the cell [3-4]. Cell fate, the stable cellular phenotype, is determined by the characteristic stable state of the complex network that is defined by a set of state values containing the activity states of all molecules in the network. Thus, systems biological investigation is crucial to understand the cell fate decision mechanism encoded in the nonlinear dynamics of the signal transduction network [5-6]. The recent study by Choi et al [7] shows that state space modeling and attractor landscape analysis of complex molecular networks can reveal a hidden cell fate decision mechanism and identify key network components that together could synergistically force cancer cells to undergo apoptosis. Choi et al investigated the regulatory mechanisms underlying p53 dynamics and its function in modulating cell fate outcome at the system level. In this study, the state transition dynamics of the p53 regulatory network were analyzed using Boolean network modeling and attractor landscape analysis where each network state is represented as a point in the cellular state space and the network state eventually converges to a fixed point (or a set of points) called the attractor state which represents the most stable state with the lowest potential energy in the state space. The analysis results revealed that the most critical network components, which determine both p53 dynamics and the cell fate outcome in response to DNA damage, are feedbacks and interactions between p53, Mdm2, Wip1, Cyclin G and ATM. Disruption of the above critical feedback controls was found to not only result in change of p53 dynamics, i.e. pulsing vs. sustained elevation, but also alteration of cell fate outcome. The attractor landscape analysis was then employed to investigate the DNA damage response of a representative breast cancer cell line, MCF7, and the effect of nutlin-3, a well-known inhibitor of Mdm2, in comparison with normal cells. Limited efficacy of nutlin-3 was indicated by its induction of small basin of attraction to apoptotic attractor in the state-space landscape with p53 dynamics mostly being oscillatory. In contrast, the analysis revealed that treatment of nutlin-3 in combination with inhibition of Wip1 would engender strong synergistic effect in activating p53-mediated apoptosis, as such combined perturbations resulted in a larger basin of attraction to apoptotic attractors with sustained elevation of p53 (Figure 1). The predicted synergistic effect was validated by single-cell imaging experiment, using a fluorescent p53 reporter line of MCF7. The experimental data suggest that a combinatorial treatment of nutlin-3 and Wip1 inhibitor is a more effective strategy for inducing apoptosis in response to DNA damaging chemotherapeutics. This study demonstrates that system-level analysis of p53 network dynamics and its regulation using attractor landscape can be employed to understand the complex cell fate decision mechanism and identify novel therapeutic strategies for treating cancer. This study also suggests a possible paradigm shift to ‘network pathology’ where we use network information instead of molecular information for personalized diagnosis and treatment of complex disease. The idea of network pathology is that the different mutation profile of each patient can be reflected in the modified network structure for individualized state space analysis. It further suggests the new concept of ‘computational chemotherapy’ that can provide an optimal therapeutic strategy for each individual patient on the basis of network pathology.
